# Phosphorylation of HPV-16 E2 at Serine 243 Enables Binding to Brd4 and Mitotic Chromosomes

**DOI:** 10.1371/journal.pone.0110882

**Published:** 2014-10-23

**Authors:** Szu-Wei Chang, Wei-Chen Liu, Kuan-Yu Liao, Yeou-Ping Tsao, Pang-Hung Hsu, Show-Li Chen

**Affiliations:** 1 Graduate Institute of Microbiology, College of Medicine, National Taiwan University, Taipei, Taiwan; 2 Department of Ophthalmology, Mackay Memorial Hospital, Taipei, Taiwan; 3 Department of Bioscience and Biotechnology, College of Life Sciences, National Taiwan Ocean University, Keelung, Taiwan; International Centre for Genetic Engineering and Biotechnology, Italy

## Abstract

The papillomavirus E2 protein is involved in the maintenance of persistent infection and known to bind either to cellular factors or directly to mitotic chromosomes in order to partition the viral genome into the daughter cells. However, how the HPV-16 E2 protein acts to facilitate partitioning of the viral genome remains unclear. In this study, we found that serine 243 of HPV-16 E2, located in the hinge region, is crucial for chromosome binding during mitosis. Bromodomain protein 4 (Brd4) has been identified as a cellular binding target through which the E2 protein of bovine papillomavirus type 1 (BPV-1) tethers the viral genome to mitotic chromosomes. Mutation analysis showed that, when the residue serine 243 was substituted by glutamic acid or aspartic acid, whose negative charges mimic the effect of constitutive phosphorylation, the protein still can interact with Brd4 and colocalize with Brd4 in condensed metaphase and anaphase chromosomes. However, substitution by the polar uncharged residues asparagine or glutamine abrogated Brd4 and mitotic chromosome binding. Moreover, following treatment with the inhibitor JQ1 to release Brd4 from the chromosomes, Brd4 and E2 formed punctate foci separate from the chromosomes, further supporting the hypothesis that the association of the HPV-16 E2 protein with the chromosomes is Brd4-dependent. In addition, the S243A E2 protein has a shorter half-life than the wild type, indicating that phosphorylation of the HPV-16 E2 protein at serine 243 also increases its half-life. Thus, phosphorylation of serine 243 in the hinge region of HPV-16 E2 is essential for interaction with Brd4 and required for host chromosome binding.

## Introduction

Papillomaviruses (PVs) are small, double-stranded DNA viruses that infect squamous epithelial cells and cause persistently infected lesions known as papillomas or warts. Human papillomavirus is one of the most common sexually transmitted pathogens in the world and has been strongly implicated in the development of anal and cervical cancer, which is the second most common cancer worldwide and the leading cause of death among women in developing countries [1]–[3]. The replication cycle of papillomaviruses is coupled to the differentiation program of the infected host keratinocytes. Infection is initiated when viruses enter the basal epithelial cells, where the viral genomes replicate to a low copy number and are maintained as extra-chromosomal episomes that replicate in synchrony with the host DNA. The viral E2 protein plays an important role in ensuring that the viral genomes are retained within the nucleus of the infected cell and are partitioned into the daughter cells during each round of cell division, by tethering the viral DNA to the host chromosomes. This mechanism helps maintain persistent infection of the host cells, so that the dividing basal cells can provide a reservoir of infected cells that migrate up to replenish the overlying, virus-producing, differentiated layers [4]–[6]. The PV E2 protein is a multifunctional protein involved in initiating viral DNA replication, regulating viral transcription and maintaining the viral genome as a replicative episome. The full-length E2 protein consists of an N-terminal transactivation domain connected by a flexible hinge region to the C-terminal dimerization and DNA-binding domain [7], [8]. Besides recruiting E1 to the viral origin to initiate PV genome replication, the E2 protein regulates viral transcription by binding to a 12-bp palindromic sequence, with a consensus motif of ACCN_6_GGT, that is present in the long control region (LCR) of the viral genome [9]. The N-terminal transactivation domain is important for the regulation of transcription and interacts with many cellular transcriptional regulatory factors, including TFIIB, NAP-1, p300/CBP, p/CAF, GR, NRIP and Brd4 [10]–[17].

Brd4 is a member of the BET family, with two conserved bromodomains that bind acetylated lysine residues of histones H3 and H4, and remains associated with mitotic chromosomes [18], [19]. Brd4 is an important partner of E2, with which it interacts; the C-terminal domain (CTD) of Brd4 being required for interaction with the bovine papillomavirus 1 (BPV-1) and human papillomavirus (HPV) E2 proteins. By associating with Brd4, BPV-1 E2 interacts with condensed chromosomes during mitosis [18]–[22] and the Brd4 CTD can act as a dominant-negative inhibitor to disrupt the interaction between E2 and Brd4, resulting in its dissociation from mitotic chromosomes [23]. In addition, it has been reported that Brd4 also is an essential component of the HPV genome replication complex and necessary for HPV-16 and HPV-31 genome maintenance during mitosis [24]–[26].

The regulatory mechanisms governing E2-mediated genome tethering and partitioning have not yet been analyzed completely. However, posttranslational modifications, such as phosphorylation, have been shown to regulate the functions of E2 proteins. Phosphorylation at the hinge regions of the BPV-1 and HPV-8 E2 proteins affects their proteasomal degradation and chromosome binding activity, respectively [27], [28]. In addition, a recent report from our laboratory revealed that HPV-16 E2 is a phosphorylated protein and changes in the E2 phosphorylation status mediated by Ca^2+^/calcineurin phosphatase through NRIP affect the protein half-life [15].

In this study, we found that two residues, S207 and S243, which lie within the hinge region of the HPV-16 E2 protein, can be phosphorylated. We have also carried out an extensive mutational analysis to verify that S243 is crucial for chromosomal association and Brd4-binding activity. Furthermore, we demonstrated that Brd4 is required for HPV-16 E2 interaction with mitotic chromosomes and dissociation of Brd4 from chromatin by the inhibitor, JQ1(+) abolishes E2 focus formation on chromosomes, supporting that E2 binding to mitotic chromosomes requires the association of Brd4 with the chromosomes [24],[25].

## Materials and Methods

### Plasmid Construction

The reporter plasmids EGFP-16E2 and LCR832 have been described previously [15]. The hinge region from positions 202 to 286 of the HPV-16 E2 protein was isolated from EGFP-16E2 by polymerase chain reaction (PCR) using specific primers containing restriction enzyme sites to construct the EGFP-16E2-HDWT expressing plasmid. The following forward and reverse primers were: 16E2-HD-F, 5′-AAA ACT CGA GCT AGC AAC GAA GTA TCC TCT CCT G-3′; 16E2-HD-R, 5′-AAA AGG ATC CTC ATG TAG TGT TAC TAT TAC AGT TAA TC-3′ (Underlining indicates restriction enzyme sites). This amplified DNA fragment was digested with XhoI/BamHI and cloned in frame into the EGFP-C1 (Clontech) vector. Amino acid substitution mutants of 16E2 and 16E2-E2HD including S207A, S243A, S207/243A, S243Q, S243E, S243N and S243D and R37A/I73A were generated from the EGFP-16E2 and EGFP-16E2-HDWT plasmids using PCR-directed mutagenesis.

### Cell culture

COS-7 is a monkey kidney fibroblast-like cell line purchased from American Type Culture Collection (ATCC; Manassas, VA, USA). HEK293 and C33A are human embryonic kidney and cervix carcinoma cell lines, respectively [15], [29]. All the cells were maintained in Dulbecco’s modified Eagle’s medium (DMEM) supplemented with 10% FBS, 1% MEM nonessential amino acids solution (Invitrogen), 2 mM L-glutamine, and antibiotics.

### Immunoprecipitation and western blotting

Cells were transfected with plasmids as indicated. At 48 h after transfection, cell lysates were harvested in NP-40 lysis buffer (300 mM NaCl, 1% NP-40, 50 mM Tris [8.0], and 1 mM phenylmethylsulfonyl fluoride [PMSF] and protease inhibitors). The lysates were subjected to sonication and centrifugation (13,000 rpm at 4°C for 15 min), and the buffer was adjusted to 150 mM salt by mixing with an equal volume of lysis buffer without NaCl. Immunoprecipitation was performed with the antibodies indicated at 4°C for 16 h. For western blotting, proteins were separated on SDS-PAGE, blotted with specific antibodies, and detected by using an ECL Western blotting detection system (Amersham Biosciences). The following antibodies were used: anti-EGFP (Clontech), anti-Brd4 (Abcam, ab128874), anti-β-actin (Sigma).

### Fluorescence microscopy assay

Human COS-7 or C33A cell lines were transfected with hyperfectin (Omics Bio), Lipofectamine 2000 Transfection Reagent (Invitrogen) or FuGENE HD Transfection Reagents (Promega) with the expression plasmid for GFP fused HPV-16 E2 proteins. For cell cycle experiments, 24 h posttransfection, cells were synchronized in 2 mM thymidine (Sigma) for 14 h. The thymidine was removed to allow progression of the cell cycle and the cells were fixed at the time points indicated. For mitotic synchronization, thymidine was removed and the cells were cultured for an additional 9 h. JQ1(+) (Bio Vision) was added during the last 3 h of each treatment. Cells were then fixed in 4% paraformaldehyde–phosphate-buffered saline (PBS) for 10 min at room temperature, permeabilized with 0.3% Triton X-100 in PBS for 10 min at room temperature and blocked with 0.5% bovine serum albumin (BSA). The Brd4 proteins were detected with monoclonal rabbit anti-Brd4 antibody (Abcam) and the slides were mounted in DAPI Fluoromount-G (SouthernBiotech). Images were collected with a Leica TCS-SP5 laser scanning confocal imaging system.

### Chromatin immunoprecipitation assays

At 48 h posttransfection, cells were fixed for 10 min with 1% formaldehyde at room temperature and lysed with lysis buffer (1% SDS, 5 mM EDTA, 50 mM Tris-HCl and protease inhibitors), centrifuged at 13000 rpm and diluted 10-fold in dilution buffer (1% Triton X-100, 2 mM EDTA, 150 mM NaCl, 20 mM Tris-HCl, pH 8.0). After overnight incubation at 4°C with the antibodies indicated, protein A/G-Sepharose beads preblocked with sheared salmon sperm DNA (Upstate) were added and incubated for further 2 h. The beads were washed sequentially in low-salt buffer, high salt buffer, LiCl buffer and Tris-EDTA buffer. The protein-DNA complexes were eluted with elution buffer (1% SDS, 0.1 M NaHCO3) and reverse cross-linked overnight at 65°C. The eluted DNA was analyzed by semiquantitative PCR using the following specific primers: (16LCR-F), 5′-CTT GCC ATG CGT GCC AAA TCC CTG-3′; (16LCR-R), 5′-GTA TGT AAG GCG TTG GCG CAT AGT G-3′.

## Results

### Phosphorylation of serine 243 targets HPV-16 E2 for chromosome binding during mitosis

**Figure 1:**
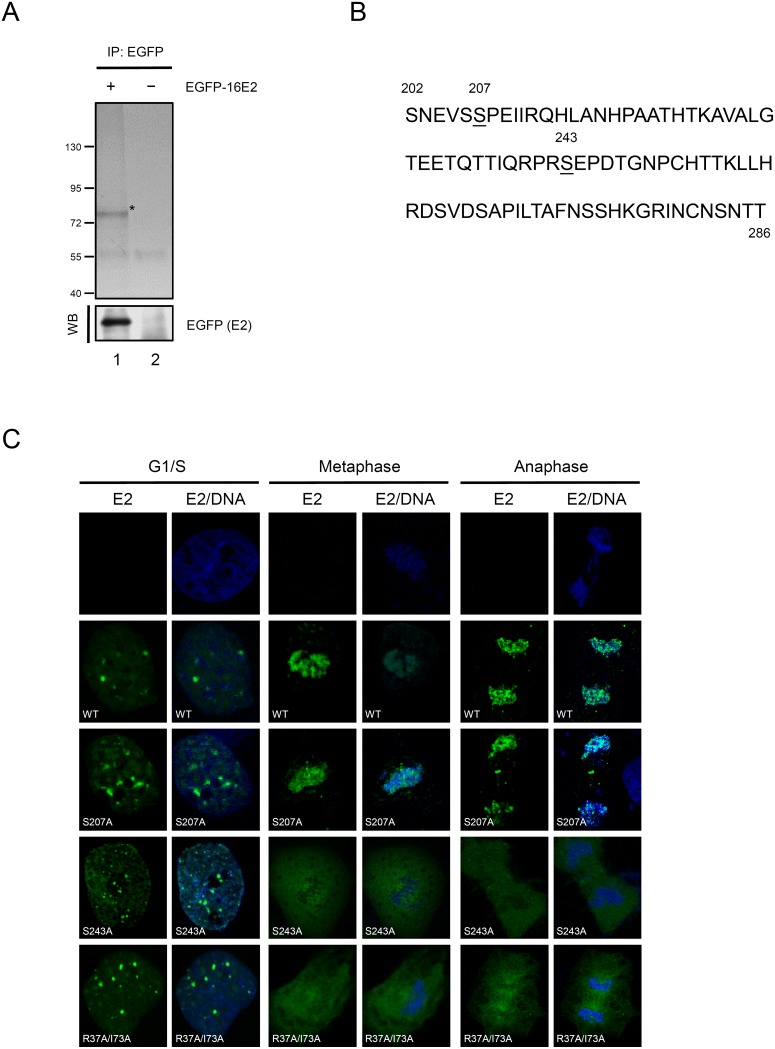
Serine 207 and 243 are potential phosphorylated residues and serine 243 mediates the chromosomal association of the HPV-16 E2 protein. A) Immunopurification and mass spectrometric analysis of phosphorylated serine on the HPV-16 E2 protein. Proteins extracted from 293 cells carrying plasmids expressing EGFP-tagged E2 proteins or the control vector were immunoprecipitated with anti-EGFP antibody in the presence of protease inhibitor (MG132). The purified lysates were resolved by SDS-PAGE followed by staining with Coomassie brilliant blue (upper) or immune-blotting with anti-EGFP antibody (lower). The visible band at size 74 kDa (marked by * in the upper panel) was cut and analyzed by mass spectrometry for the phosphorylated residues. B) HPV-16 E2 proteins are phosphorylated at serine 207 and 243. Amino acids 202 to 286 from the hinge region of HPV-16 E2 are shown and the both potential phosphor-serines are underlined. C) Chromosome associating phenotypes of the E2 proteins with mutations in the hinge at G1/S, metaphase and anaphase. Localization of EGFP-tagged HPV-16 E2 proteins, including with substitutions of serine 207, serine 243 and R37A/I73A, in mitotic COS-7 cells is shown in green and DNA was stained with DAPI (blue) and visualized by confocal microscopy.

We have previously shown that the HPV-16 E2 protein is phosphorylated and the phosphorylation status affects its half-life [15]. To begin searching for the phosphoacceptor residues of the E2 protein, the phosphorylation status of E2 was analyzed using the proteasomal inhibitor MG132 to block its degradation. Treatment with MG132 results in the accumulation of the rapidly turned-over E2 protein and the accumulated phosphorylated E2 can be analyzed. These experiments were carried out using 293 cells transfected with expression vectors for EGFP-tagged HPV-16 E2 proteins or mock transfected. The E2 proteins were purified using an anti-EGFP antibody and subjected to phosphorylation analysis by MALDI-TOF (Fig. 1A, lane 1). Based on the results of MALDI-TOF analysis, we found two phosphoacceptor residues at serine 207 and serine 243 in the hinge region, which is located from positions 202 to 286 in the HPV-16 E2 protein (Fig. 1B; Fig. S1). Unlike the N-terminal transactivation and C-terminal DNA binding domains, the hinge region of E2 proteins is not well conserved among the papillomaviruses [30]. However, it has been reported that the hinge is required for the association of E2 with mitotic chromosomes [4], [31] and phosphorylation regulates binding of the E2 protein to the host chromosomes [27]. To elucidate whether these two residues are critical for chromosome binding by the HPV-16 E2 protein, we substituted each of these serine residues with alanine (named S207A and S243A) in the background of the full-length HPV-16 protein, to eliminate phosphorylation, and examined the localization of these E2 proteins on the mitotic chromosomes by confocal microscopy. As shown in Fig. 1C, alanine substitutions of residue S207 or S243 exhibited the wild type pattern of nuclear localization in interphase cells (G1/S) and the proteins could form speckles, as does the wild type E2. During metaphase and anaphase, wild type E2 and S207A could bind with the mitotic chromosomes, but S243A was excluded from the condensed chromosomes and anaphase chromosomes and was located in the cytosol that was similar to well-known mutant 16E2 R37A/I73A [30]. This indicates that S243A is deficient for mitotic binding and does not form mitotic chromosomal foci. Hence, from this study, we can conclude that we discovered two potential serine residues that can be phosphorylated in the hinge region of the HPV-16 E2 protein but only serine 243 is crucial for chromosomal binding during mitosis.

### Serine 243 mediates binding to Brd4

**Figure 2:**
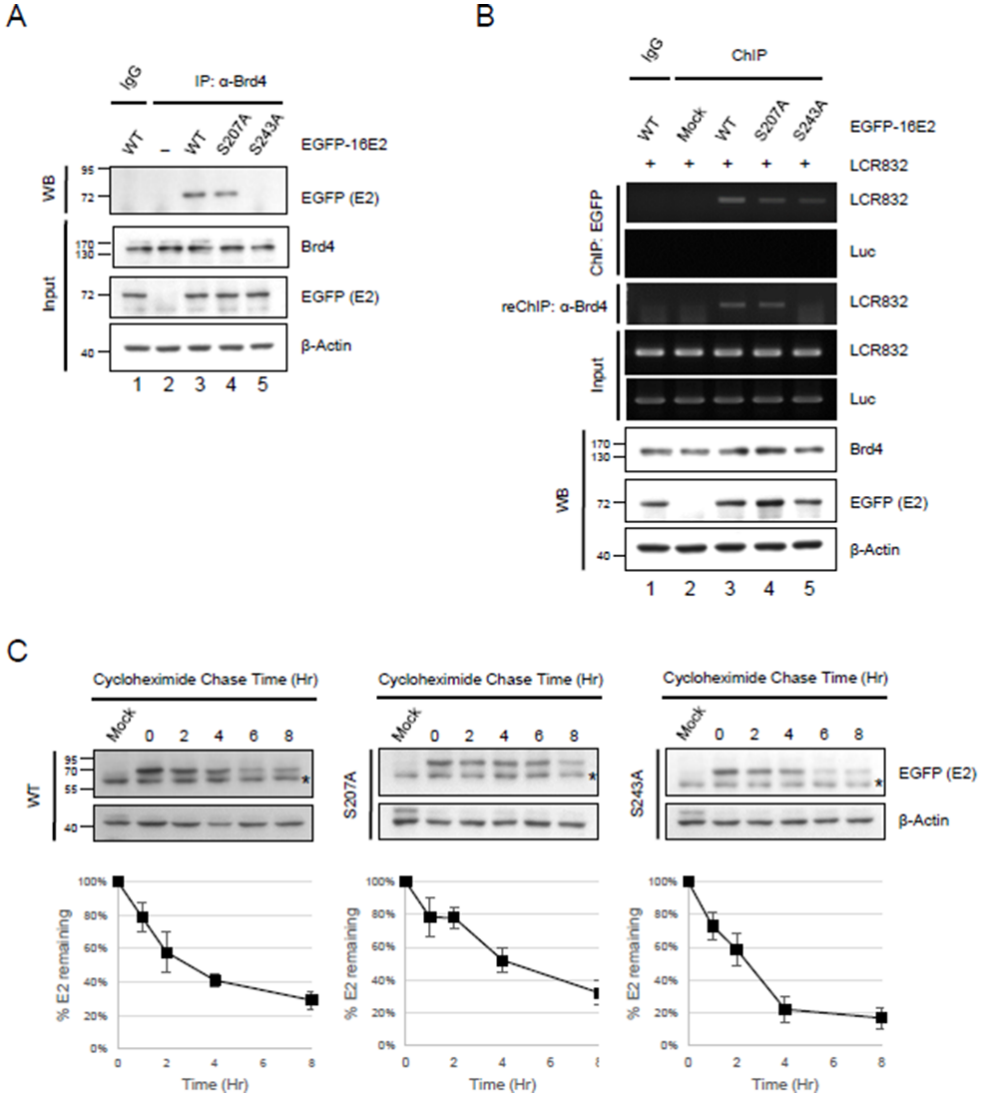
Residue serine 243 is critical for the association of HPV-16 E2 with Brd4. A) 293 cells were transfected with expression vectors for EGFP-tagged HPV-16 E2 or mutated E2. Cell extracts were immunoprecipitated with anti-Brd4 antibody or anti-IgG as a control in incubation buffer containing EtBr and the immunoblotting was performed with antibodies as indicated. B) C33A cells were cotransfected with HPV-16 LCR reporter and EGFP-tagged E2 or mutants. At 48 h posttransfection, chromatin extracts were immunoprecipitated with antibody against Brd4. The eluted DNA-protein complexes were subjected to PCR analysis (ChIP) or further immunoprecipitation with anti-Brd4 antibody (reChIP). C) The S243-mutated E2 protein (S243A) has a shorter half-life than the wild type. 293 cells were transfected with EGFP-tagged wild type or mutated E2. At 36 h posttransfection, the cells were treated with cycloheximide (50 µg/ml) for up to 8 h. Cell extracts were collected at the times shown and immunoblotted with the antibodies indicated. The graph below presents the quantified intensities of the bands. The expression levels of E2 were normalized using β-actin and the percentage of E2 at each time point relative to that of the control (without CHX treatment, set at 100) are shown. Data shown are means of results from three independent experiments (mean ± standard deviation; n = 3).

E2 plays an important role in establishing persistent infection and an essential feature of its function is its ability to tether the viral genomes to host chromosomes during mitosis in order to ensure their retention in the nucleus and partitioning during cell division [32]. Furthermore, it has been shown that Brd4 interacts with the E2 proteins of most papillomaviruses on mitotic chromosomes to mediate tethering of the viral genome [20], [24], [25]. We then determined whether phosphorylation at S243 of the HPV-16 E2 protein regulates the binding activity to Brd4 using coimmunoprecipitation assays. HEK293 cells were transfected with the expression vectors for EGFP-tagged HPV-16 E2, S207A or S243A mutant proteins and the cell lysates were immunoprecipitated with anti-Brd4 antibody in an incubation buffer containing EtBr to avoid DNA interference. The immunoprecipitated protein complexes were analyzed by SDS-PAGE and western blotting using antibody against EGFP (E2). Fig. 2A shows that only the S243A mutant protein was defective in binding to Brd4 (lane 5), and not the wild type E2 and S207A (lanes 3 and 4) or wild type E2 with IgG precipitates (lane 1). This indicates that phosphorylation at S243 of E2 is able to regulate binding to Brd4. To investigate further the formation of the DNA tethering complex E2:Brd4 in the cells, the expression vectors for wild type or mutant E2 proteins were cotransfected with the HPV-16 long control region (LCR832) plasmid, containing four E2-binding sites, into C33A cells. EGFP-tagged E2 proteins were immunoprecipitated, along with the HPV-16 promoter, by anti-EGFP antibody, followed by a second round with antibody against Brd4 to immunoprecipitate the DNA-protein complex. As shown in Fig. 2B, wild type E2 bound to the HPV-16 LCR promoter (lanes 3) and S207A and S243A also could bind to the HPV promoter, but to a lesser extent than wild type E2 (lane 4 and 5). However, in a re-ChIP assay for Brd4 binding, the S243A mutant had lost the ability to bind Brd4 (lane 5), whilst wild type E2 and S207A could bind to Brd4 and DNA to form a DNA-tethering complex (lane 3 and 4). This result is consistent with Fig. 2A, indicating that serine 243 in the HPV-16 E2 protein is a key residue in regulating Brd4 binding. Because Brd4 binding can increase the stability of the E2 protein [33], [34] and it also has been reported that the E2 protein is unstable when not bound to the chromosomes [27], we carried out a pulse-chase experiment and compared the half-life of the S207A and S243A mutants to the wild type E2 protein. HEK293 cells were transfected with the expression vectors for wild type, S207A or S243A-mutated E2 proteins for 36 h and then treated with cycloheximide to block *de* *novo* protein synthesis. Cell extracts were collected at the times indicated after cycloheximide treatment and the E2 protein levels were analyzed by western blotting. Previous reports have shown that the half-life of HPV-16 E2 is about 1 h [30], in our system wild type E2 has a half-life of around 3 h in 293 cells [15]. Similarly, the wild type E2 protein exhibited a 3 h half-life (Fig. 2C, panel a), whilst the half-lives of the S207A and S243A proteins were 4 h and 2 h, respectively (Fig. 2C, panel b and c). In summary, the S243A E2 protein has a shorter half-life than the wild type and S207A E2, indicating that the HPV-16 E2 protein phosphorylated at serine 243 increases its half-life.

### Phosphorylation at serine 243 is crucial for Brd4 binding

**Figure 3:**
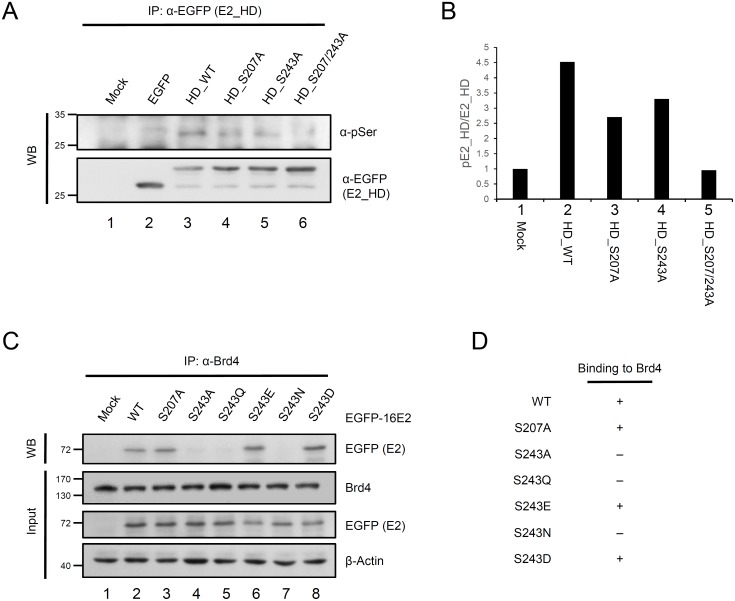
Phosphorylation at serine 243 of HPV-16 E2 mediates binding to Brd4. A) Phosphorylation analysis and HPV-16 E2 proteins. 293 cells were transfected with expression vectors for wild type or mutated hinge region of HPV-16 E2 fused to EGFP. Equivalent amounts of protein lysate were immunoprecipitated using anti-EGFP antibody and the samples were analyzed by western blotting with antibody against phosphor-serine. B) The graph presents the quantified intensities of the phosphorylated signal. The level of phosphorylated E2 hinge was normalized using total E2 immunoprecipitated. C) Mutational analysis of E2 proteins binding to Brd4. 293 cells were transfected with vectors expressing various E2 proteins containing amino acid substitutions in the hinge region. Cell lysates were collected and immunoprecipitated with anti-Brd4 antibody. The protein complexes were analyzed by SDS-PAGE and subjected to immunoblotting with anti-EGFP antibody. The expression level of E2 proteins was normalized using β-Actin, and the percentage of E2 at each time point relative to that of the control (without CHX treatment, set at 100) is shown (lower panel) Data shown are means of results from three independent experiments. Asterisk, non-specific band. D) Summary of the Brd4-binding activity of the HPV-16 E2 proteins and mutants.

To confirm that serine 243 in HPV-16 E2 is a phosphorylation site, the plasmid DNAs, including EGFP-tagged hinge domains from positions 202 to 286 and containing serine residues substituted with alanine to exclude potential phosphorylation sites in other regions, were constructed as described in Materials and Methods. These E2 protein fragments were expressed in 293 cells, immunoprecipitated from equivalent counts of total protein with anti-EGFP antibody and subjected to analysis by gel electrophoresis, followed by western-blotting, using antibody against phosphor-(Serine). Fig. 3A shows that the wild type hinge region of the E2 protein was detectable using anti-phosphor-(Serine) antibody (lane 3), indicating that the hinge is phosphorylated at serine residues. Notably, there was a substantial reduction of signals from the S207A (lane 4) and S243A proteins (lane 5) and the hinge protein containing two amino acid substitutions (S207/243A) exhibited no detectable phosphorylation (Fig. 3A, lane 5). The calculation of band intensity from Fig. 3A is shown in Fig. 3B and confirms that both serine residues 207 and 243 are phosphorylation sites in the hinge region of the HPV-16 E2 protein, as shown in Fig. 1B. However, S207A was able to associate with mitotic chromosomes, indicating that phosphorylation at serine 207 of the E2 protein is not required for tethering to host chromosomes during mitosis (Fig. 1C panel b and c). To analyze further the contribution of phosphorylation of the serine 243 residue to the mitotic chromosomal binding through binding to Brd4, a set of substitutions was generated at residue position 243, as described in Materials and Methods. The residue serine 243 was substituted by glutamic acid (E) and aspartic acid (D) (named S243E and S243D respectively), which are negatively charged residues that mimic the effect of constitutive phosphorylation, or polar uncharged residues such as asparagine (N) and glutamine (Q) (named S243N and S243Q respectively). The expression plasmids for all mutated E2 proteins were introduced into 293 cells, followed by immunoprecipitation and western blotting using anti-Brd4 and anti-EGFP (E2), respectively. According to the results in Fig. 3C, E2 proteins capable of phosphorylation at serine 243, including the phosphor-mimetic substitutions S243D and S243E, were able to interact with Brd4 (lanes 6 and 8), while E2 proteins with S243A, S243Q and S243N lost the ability to bind to Brd4 (Fig. 3C, lanes 5 and 7). The data in Fig. 3C are summarized in Fig. 3D. Therefore, we can conclude that phosphorylation at serine 243 in the hinge region is essential for interaction with Brd4.

### The residue serine 243 of the HPV-16 E2 protein is essential for chromosomal binding

**Figure 4:**
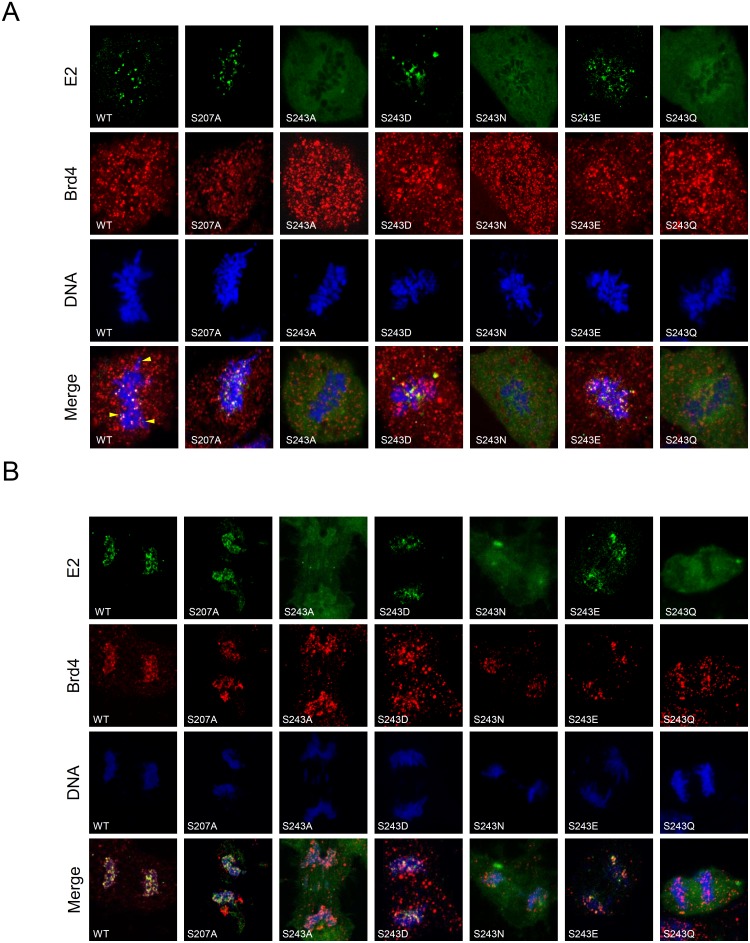
Chromosome association phenotypes of Brd4 and E2 proteins with amino acid substitutions in the hinge. A, B) Phosphorylation at residue 243 is critical for the co-localization of HPV-16 E2 and Brd4 and association with mitotic chromosomes. COS-7 cells transfected with plasmids expressing HPV-16 wild type E2, S207-mutated E2 (S207A) or S243-mutated E2 (S243A, S243D, S243N, S243E, S243Q) were visualized by confocal microscopy to determine the location of E2 (green), Brd4 (red) and cellular DNA (blue) during mitosis. A) metaphase and B) anaphase.

Based on the data in Fig. 1C, Fig. 2A and Fig. 3A, we found that phosphorylation at S243 is either critical for chromosomal association or important for E2 binding to Brd4. Brd4 is a member of the BET family that contains bromodomains that bind acetylated histones and is associated with the chromosomes during mitosis [19]. We then examined the localization of E2 and Brd4 in mitotic cells. COS-7 cells were transfected with the expression vectors for the E2 proteins, including the wild type and proteins substituted at S207 or S243. The E2 and Brd4 protein expression in cells was shown by immunoblotting (Fig. S2), and the colocalization of the E2 mutants and Brd4 during mitosis was investigated by confocal microscopy. A negative control (GFP expression alone) was done to demonstrate Brd4 localization during cell cycle and shown in Figure S3. It indicates that Brd4 location was not affected by GFP expression in interphase, metaphase and anaphase. Additionally, Figure S3 showed the each mutated E2 protein locates in nucleus in interphase cells. Fig. 4A and 4B show that both the wild type and S207A E2 proteins were associated with mitotic chromosomes and colocalized with Brd4 in metaphase and anaphase. We observed E2 alone, not colocalized with Brd4 (marked by a yellow arrow), located in the condensed chromosomes, indicating that there are other ways for E2 to interact with the mitotic chromosomes. Meanwhile, the E2 mutants, such as S243A, S243N and S243Q, which are defective in Brd4 binding (Fig. 3C), could not form punctate nuclear speckles and colocalize with Brd4 in the condensed metaphase (Fig. 4A) and anaphase (Fig. 4B) chromosomes. However, the phosphor-mimetic substitution E2 mutants S243D and S243E associated with Brd4 and the metaphase (Fig. 4A) and anaphase (Fig. 4B) chromosomes, as does the wild type E2. To quantitate chromosome binding for each of the mutated proteins, approximately 70–140 mitotic cells from each cell line were analyzed. Table 1 shows the number of mitotic cells examined, the proportion of E2 expressing cells, and percentage of E2-positive cells with E2 foci located on mitotic chromosomes. In summary, phosphorylation at residue 243 in hinge region of HPV-16 E2 protein is required for binding to the chromosomes and Brd4.

**Table 1:** E2-expressing mitotic cells showing the focus binding pattern shown in Fig. 4.

E2 protein	No. of mitotic cellsexamined	% Cells expressingE2	% E2-positive cells with E2 focilocated on mitotic chromosomes
WT	122	28.6	74.6
S207A	86	30.8	75.6
S243A	133	33.9	19.5
S243D	105	33.6	65.7
S243E	78	27.9	70.5
S243N	83	23.4	14.5
S243Q	80	24.2	20.0

### Brd4 is necessary for HPV-16 E2 association with mitotic chromosomes

**Figure 5:**
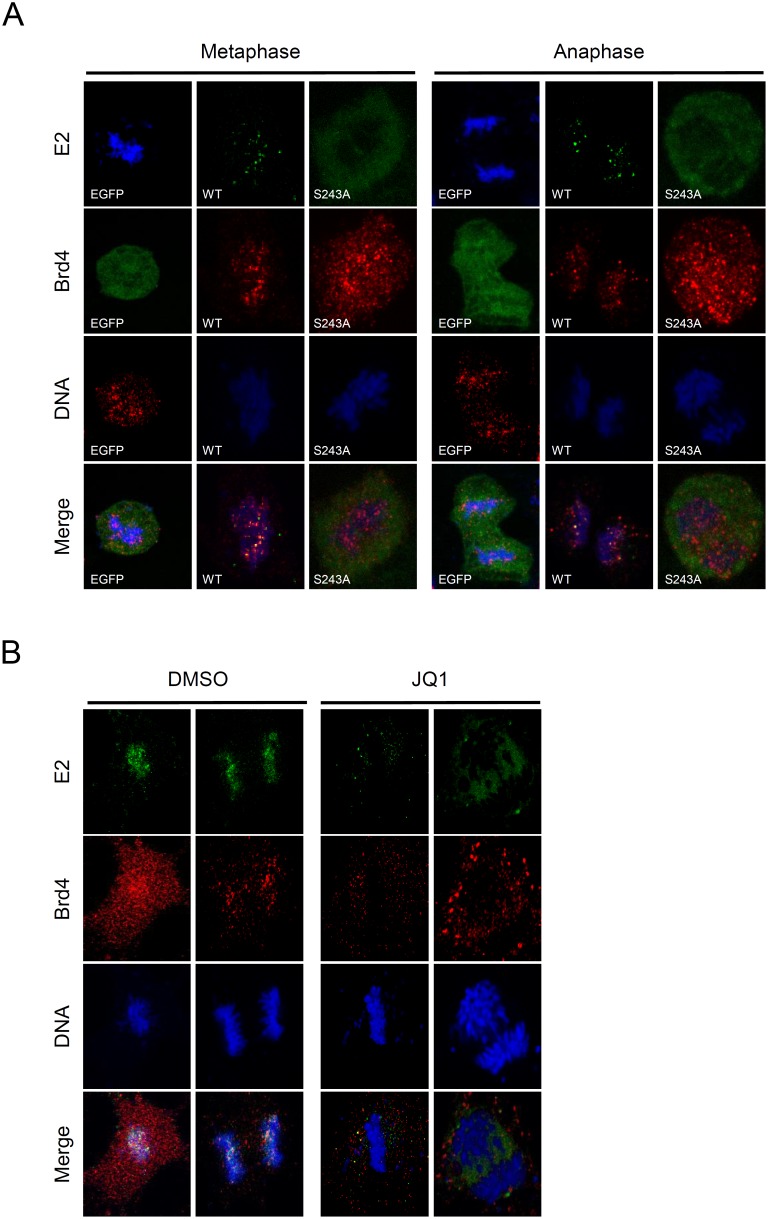
HPV-16 E2 binds to the chromosomes in a Brd4-dependent manner. A) C33A cells were transfected with the expression plasmids for EGFP-fused HPV-16 wild type, S243A E2, or EGFP proteins. After fixation, the cells were subjected to fluorescence microscopy after counterstaining of the DNA with DAPI. The cells in metaphase (*left*) and anaphase (*right*) are shown. B) Inhibition of Brd4 chromatin binding causes E2 to dissociate from the chromosomes during mitosis. COS-7 cells were transfected with EGFP-tagged E2 and treated with DMSO or 0.5 µM JQ1(+). Location of protein complexes was detected by confocal microscopy for E2 (green), Brd4 (red) and DNA (DAPI).

Our results show that the E2 protein is predominantly colocalized with Brd4 during metaphase and anaphase (Fig. 4). In this study, Brd4 showed a dispersed distribution throughout the entire COS-7 cell at metaphase (Fig. 4A) and this phenotype differed from observations made in C33A cells, in which Brd4 is located in the condensed chromosomes [18], [19], [35]. However, at late anaphase and telophase, when the sister chromatids are pulled apart into the daughter cells, Brd4 was found to mainly colocalize with the chromosomes (Fig. 4B), which is similar to HeLa and C127 cells [36]. We then asked whether HPV-16 E2 binding Brd4 resulted in the colocalization to the condensed metaphase chromosomes in cells other than COS-7. C33A cells were used to express the wild type or S243A mutant E2 proteins and, as shown in Fig. 5A, the wild type E2 proteins associated with Brd4 and were colocalized with the chromosomes throughout mitosis, but the S243A mutant E2 was not, which is consistent with the observation in COS-7 cells (Fig. 4A). Similarly, Brd4 was concentrated in the condensed chromosomes, as in previous reports [18], [19], [35]. Moreover, to elucidate whether the association of the HPV-16 E2 protein with the chromosomes is Brd4-dependent, we treated the cells with JQ1(+), which inhibits Brd4 binding to chromosomes, resulting in the effective release of Brd4 from chromatin. COS-7 cells were then transfected with the EGFP-16E2 construct and treated with 500 nM JQ1 or solvent for 30 minutes prior to confocal microscopy. After JQ1(+) treatment, Brd4 and E2 formed punctate foci dissociated from the chromosomes (Fig. 5B), confirming that HPV-16 E2 binding to mitotic chromosomes requires the association of Brd4 with the chromosomes.

## Discussion

In this study, we found that the serine 243 of HPV-16 E2, located in the hinge region, is crucial for chromosomal binding during mitosis. Because there are two potential phosphorylation sites at S207 and S243 (Fig. 1B), we investigated two substituted proteins, S207A and S243A. During interphase, both proteins showed an even distribution in nucleus, as does the wild type (Fig. 1C). However, during metaphase and anaphase, S243A remained located in the cytosol, whilst the wild type and S207A were located in the mitotic chromosomes (Fig. 1C). Moreover, mutation analysis showed that S243D and S243E can interact and colocalize with Brd4 in condensed metaphase and anaphase chromosomes, but S243Q and S243N cannot, because the substituted amino acids E and D are negatively charged residues that mimic the effect of constitutive phosphorylation, whilst the polar uncharged residues N and Q did not enable binding to Brd4 and mitotic chromosomes (Fig. 4). This indicates that the phosphorylation is essential for binding to Brd4 and mitotic chromosomes. Furthermore, following treatment with the inhibitor (JQ1) to release Brd4 from the chromosomes, Brd4 and E2 formed punctate foci dissociated from the chromosomes (Fig. 5B), supporting the hypothesis that association of the HPV-16 E2 protein with the chromosomes is Brd4-dependent [25].

Previously, it has been difficult to detect by immunofluorescence the colocalization of the E2 proteins from the alpha-PVs (such as HPV-11, 16, 31, 57) with Brd4 on metaphase and anaphase chromosomes, although they could be detected bound to host chromosomes in CV1 and C33A cells at the beginning (prophase) and end (telophase) of mitosis [16], [37], [38]. By using biomolecular fluorescence complementation technology, the interaction between HPV-16 E2 and Brd4 could be detected both on interphase chromatin and mitotic chromosomes in the entire mitosis process [25]. However in this study, we observed E2-Brd4 foci in metaphase, and more clearly in anaphase. This may be attributable to the use of a different cell line or different Tag for E2 protein expression; here we used wild type and mutant E2 tagged with EGFP at the N terminus and expressed in COS-7 cells. This may suggest that cellular factors other than Brd4 may be involved in focus formation during mitosis.

The N terminal domain of papillomavirus E2 is responsible for Brd4 binding and the amino acids R37 and I73 in this region of BPV-1 E2 are responsible for Brd4 binding to mediate the association with the mitotic chromosomes. Mutation of amino acids R37 and I73 of BPV-1 E2 disrupts the interaction with Brd4, resulting in the loss of binding to the mitotic chromosomes [16], [20], [39], [40]. In this study, we have identified a new binding site for Brd4, the residue S243 located at the hinge region of HPV-16 E2. The single-amino-acid substitutions of S243 to alanine or positively charged amino acids caused the loss of Brd4 binding so that these substituted E2 proteins could not bind to the mitotic chromosomes. Our data show that S243 phosphorylation is a key factor of HPV-16 E2 binding to the mitotic chromosomes in physiologic conditions. However, previous *in* *vitro* studies showed that E2 protein expressed and purified from *E. coli*, can still bind to Brd4 [17], [24], suggesting that E2 phosphorylation is not required for the interaction. Due to *in* *vitro* binding can be occurred in buffer ionic strength and high protein expression amount. However, the S243 phosphorylation in the hinge region may be necessary to keep structure of E2 protein accessible to the Brd4 binding in physiological condition.

The papillomavirus E2 protein helps maintain persistent infection of the host cells by partitioning the viral genome to the daughter cells during cell division [30]. Thus, the E2 protein is responsible for the association with the mitotic chromosomes but the mitotic binding pattern varies among the E2 proteins from different genera. BPV-1 E2 binds to host chromosomes as small speckles over the arms of all mitotic chromosomes, in a complex with the cellular protein Brd4 [18], [21], whilst the HPV-8 E2 protein shows a distinct pattern of large foci bound to the pericentromeric regions of the chromosomes [4], [31]. Our results show that the distribution of HPV-16 E2-Brd4 speckles in the arms of the host chromosomes is similar to BPV-1 E2. However, we also detected the localization of E2 in the mitotic chromosomes without its association with Brd4 (Fig. 4). Thus, we cannot rule out that E2 may bind directly to the host chromosomes or interact with other cellular proteins, such as ChIR1 (an ATP-dependent DNA helicase important for sister chromatid cohesion) [41], the mitotic spindle [42], a mitotic kinesin like protein, MKlp2 [43], and TopBP1 [37], for viral genome segregation. In addition, the mutated 173L E2 protein of HPV-31 cannot bind to Brd4 but still maintains extrachromosomal viral genomes and undergoes amplification in differentiated keratinocytes [44]. Hence, the involvement of HPV-16 E2 in chromosome segregation may be both Brd4-dependent and –independent. On the other hand, Brd4 stabilizes E2 by blocking the ubiquitination at its N terminus to protect against proteasome binding [30], [33], [45] and the hinge region of E2 serving as a flexible structure can prevent proteasomal degradation in BPV-1 E2 [46]. Hence our study showed that S243 located in the hinge region responsible for Brd4 binding may also plays a role for E2 protein stabilization.

Brd4 is a double bromodomain-containing protein and bromodomains preferentially bind to acetylated chromatin during cell growth and for cell cycle regulation [47]. Association of the C terminal domain of Brd4 with the N-terminal domain of E2 induces a conformational change that allosterically enhances the C-terminal DNA binding activity of E2 to its cognate sequences in the promoter region of the HPV genome [4]. Brd4 has been shown to associate with mitotic chromosomes at all stages of mitosis [19]. An interesting observation in this study was that Brd4 shows an evenly dispersed distribution in COS-7 cells at metaphase (Fig. 4A) and this phenotype differs from the observations made in other cell lines (such as C33A), in which Brd4 can form speckles in metaphase chromosomes [18], [19], [35]. However, in COS-7 cells during late anaphase and telophase, when the sister chromatids are pulled apart into the daughter cells, Brd4 was found to colocalize predominantly with the chromosomes (Fig. 4B), which is similar to HeLa and C127 cells [36]. In similar experiments in C33A cells, E2-Brd4 foci were detectable in both metaphase and anaphase chromosomes (Fig. 5A). This indicates that, regardless of Brd4 localization/distribution, HPV-16 E2-Brd4 foci (speckles) can be detected in metaphase and anaphase in both COS-7 and C33A cells.

Our data show the importance of the S243 residue, which is located in the consensus RXXS domain and equivalent to S253 in the RXXS motif of the hinge region in HPV-8 E2 but not conserved in other alpha HPVs. This serine 253 residue is known to be required for mitotic chromosome binding [4], [27]. The phosphorylation of S253 is required for direct interaction with mitotic chromosomes in the absence of Brd4. However, substitution of D for S253 abrogates binding to the mitotic chromosomes, and this differs from our data showing that HPV-16 S243D could bind to Brd4 and form foci (speckles) in metaphase and anaphase chromosomes (Fig. 4). Our data show that phosphorylation of S243 in the RXXS motif or its substitution with a negatively charged amino acid enables interaction with Brd4 and the formation of foci in condensed chromosomes and during anaphase. It may be that a negative charge, including through phosphorylation, induces a conformational change enabling the E2 protein to interact with Brd4 and bind to mitotic chromosomes.

In summary, we found that serine 243 of HPV-16 E2, located in the hinge region, is crucial for chromosomal binding during mitosis. The association of the HPV-16 E2 protein with the chromosomes is Brd4-dependent. In addition, the S243A substituted E2 protein has a shorter half-life than the wild type, indicating that the HPV-16 E2 protein phosphorylated at serine 243 plays a role in interacting with Brd4 and this increases the protein half-life. Thus the phosphorylation at serine 243 in the hinge region of HPV-16 E2 is essential for interaction with Brd4 and is required for host chromosome binding.

## Supporting Information

Figure S1 **Identification of Ser207 and Ser243 as the targets of phosphorylation by mass spectrometry.** The E2 protein of HPV-16 was purified from 293 cells. The coomassie blue-stained purified protein bands were performed in-gel trypsin digestion followed by the LC-MS/MS analysis. The CID-MS/MS spectra of phosphopeptides containing Ser207 (SSNEVSpSPEIIR) and Ser243 (pSEPDTGNPCHTTK) were demonstrated.
(TIF)

Figure S2 **Protein expression levels of Brd4 and E2 with amino acid substitutions in the hinge.** COS-7 cells were transfected with expression vectors for HPV-16 wild type E2, S207-mutated E2 (S207A) or S243-mutated E2 (S243A, S243Q, S243E, S243N, S243D). Cell lysates were collected and analyzed by western blotting with anti-Brd4 and anti-EGFP antibody.
(TIF)

Figure S3 **The localization phenotypes of E2 proteins with amino acid substitutions in the hinge in interphase cells.** COS-7 cells expressing wild type E2 (WT) or E2 proteins with amino acid substitutions in the hinge, or mock transfection (EGFP) were assayed for E2 and Brd4 localization in interphase cells by immunofluorescence. The EGFP-tagged E2 is shown in green; the Brd4, as detected by a Texas Red antibody, is shown in red; cellular DNA was stained with DAPI (blue).
(TIF)
