# Supplementary figures and images for: Phosphorylation of HPV-16 E2 at Serine 243 Enables Binding to Brd4 and Mitotic Chromosomes
Source: PLoS One. 2014 Oct 23;9(10):e110882. doi: 10.1371/journal.pone.0110882 (PMC4207782; doi:10.1371/journal.pone.0110882)

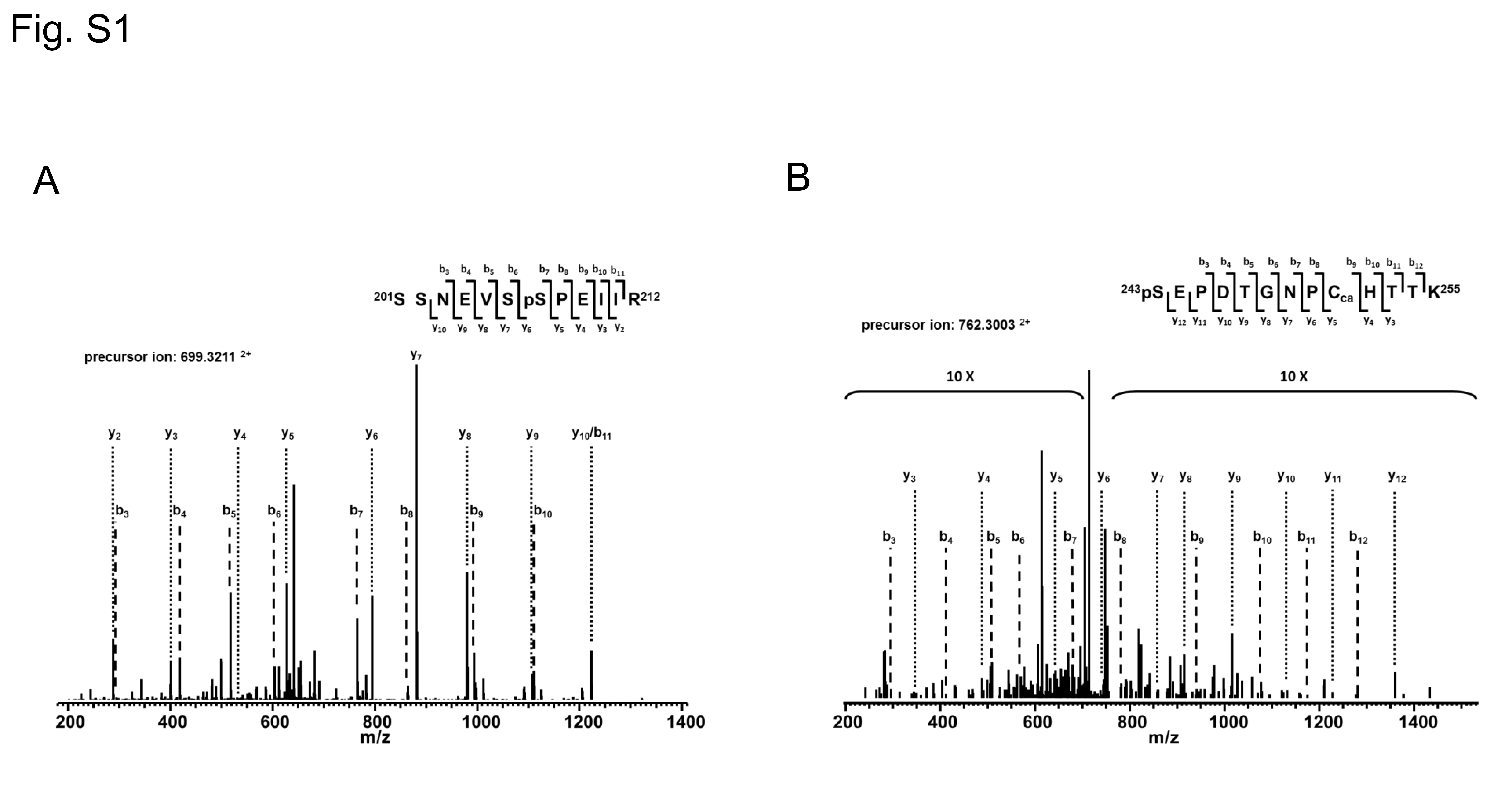

Supplement: Figure S1 — Identification of Ser207 and Ser243 as the targets of phosphorylation by mass spectrometry. The E2 protein of HPV-16 was purified from 293 cells. The coomassie blue-stained purified protein bands were performed in-gel trypsin digestion followed by the LC-MS/MS analysis. The CID-MS/MS spectra of phosphopeptides containing Ser207 (SSNEVSpSPEIIR) and Ser243 (pSEPDTGNPCHTTK) were demonstrated. (TIF) [file pone.0110882.s001.tif]

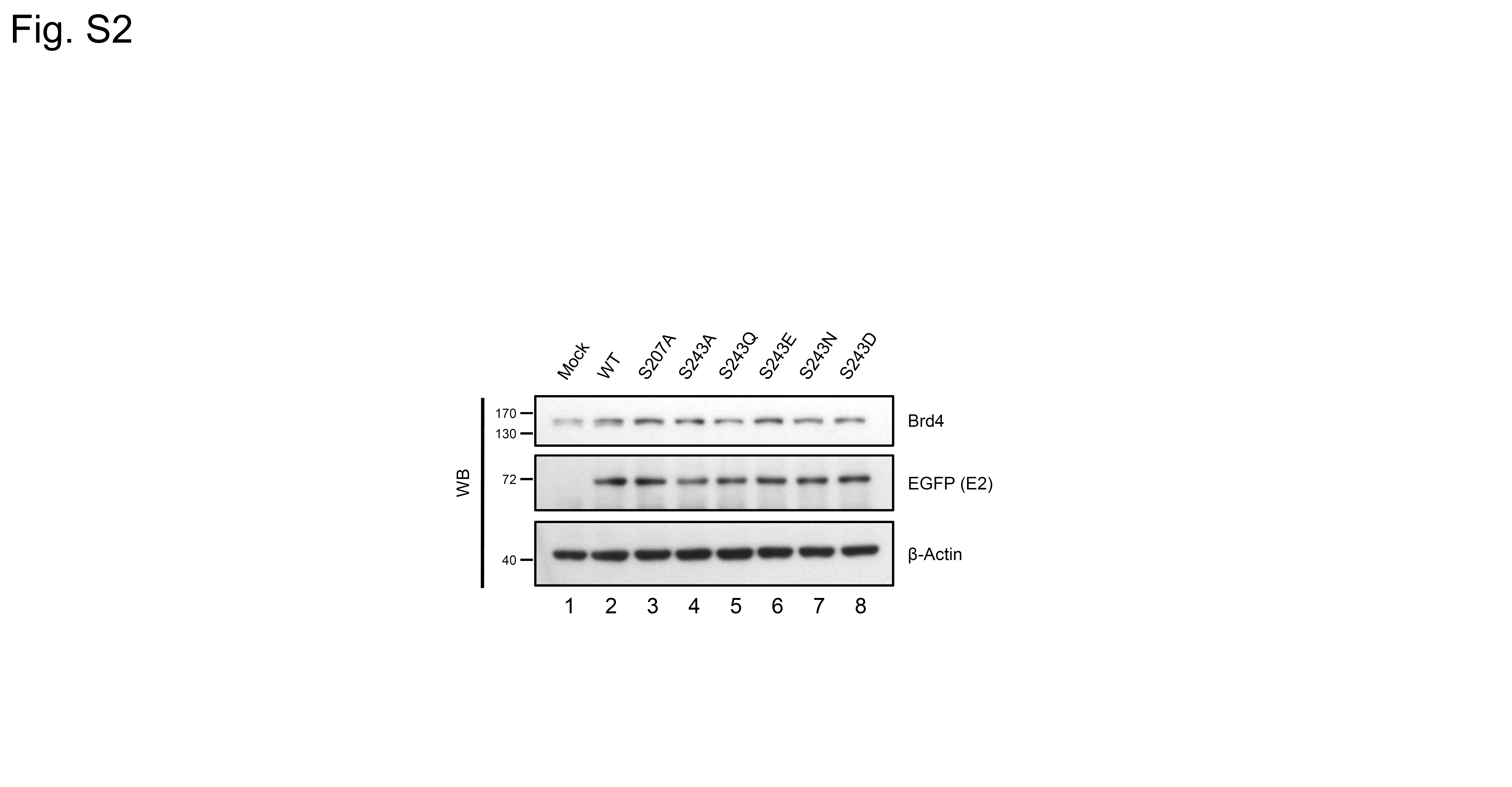

Supplement: Figure S2 — Protein expression levels of Brd4 and E2 with amino acid substitutions in the hinge. COS-7 cells were transfected with expression vectors for HPV-16 wild type E2, S207-mutated E2 (S207A) or S243-mutated E2 (S243A, S243Q, S243E, S243N, S243D). Cell lysates were collected and analyzed by western blotting with anti-Brd4 and anti-EGFP antibody. (TIF) [file pone.0110882.s002.tif]

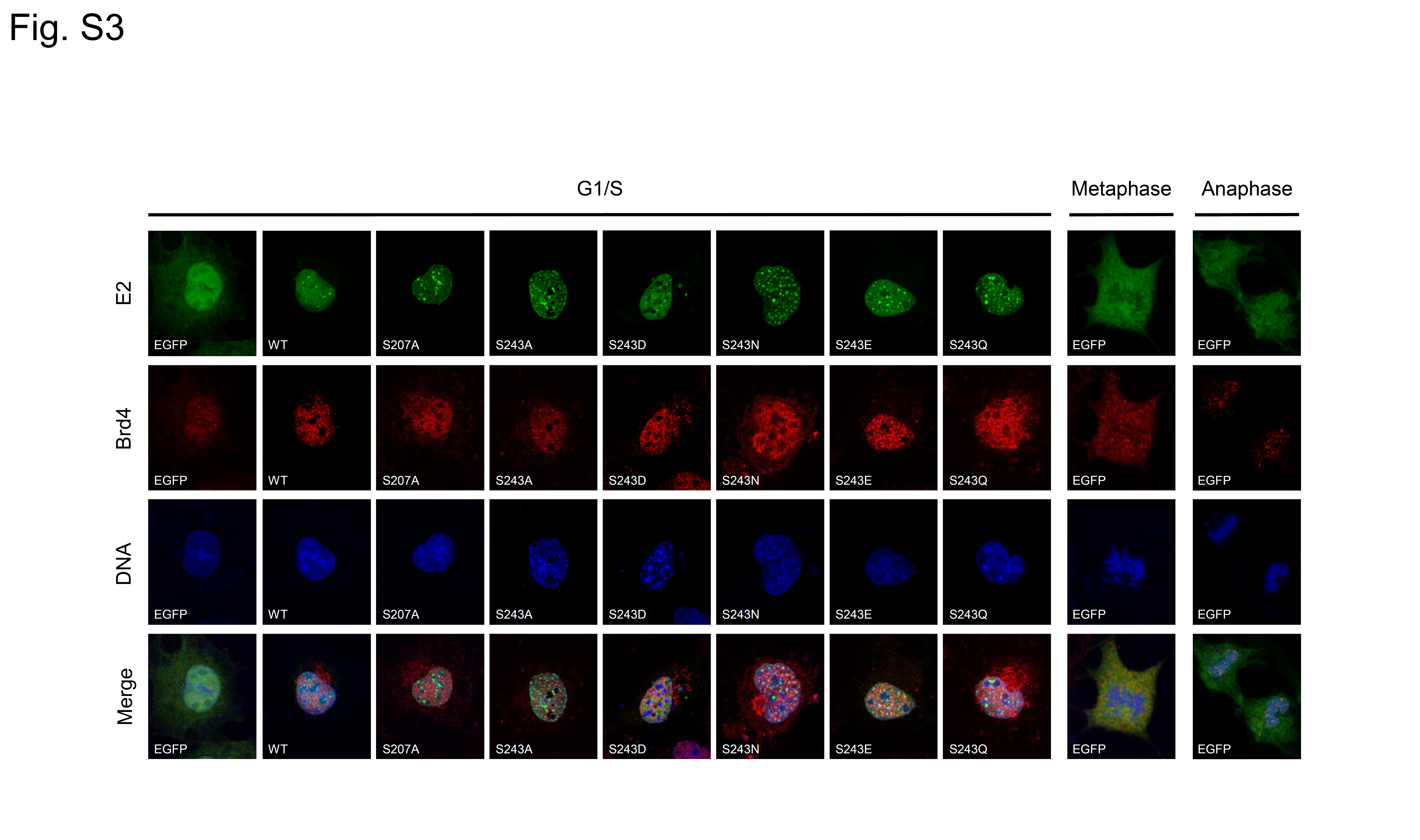

Supplement: Figure S3 — The localization phenotypes of E2 proteins with amino acid substitutions in the hinge in interphase cells. COS-7 cells expressing wild type E2 (WT) or E2 proteins with amino acid substitutions in the hinge, or mock transfection (EGFP) were assayed for E2 and Brd4 localization in interphase cells by immunofluorescence. The EGFP-tagged E2 is shown in green; the Brd4, as detected by a Texas Red antibody, is shown in red; cellular DNA was stained with DAPI (blue). (TIF) [file pone.0110882.s003.tif]
